# Supplementary material for: Global disparities in the introduction, scale-up, and effectiveness evaluation of COVID-19 vaccines
Source: Nat Commun. 2025 Oct 13;16:9059. doi: 10.1038/s41467-025-63950-w (PMC12518578; doi:10.1038/s41467-025-63950-w)
Supplement: Supplementary file 1 — Supplementary Information [file 41467_2025_63950_MOESM1_ESM.pdf]

Supplementary Appendix

Global disparities in the introduction, scale-up, and effectiveness evaluation of COVID-19 vaccines

Martina Pesce, Daniel R. Feikin, Melissa M. Higdon, Katherine L. O'Brien, Minal K. Patel, Analía Rearte, Carla Vizzotti, Annelies Wilder-Smith, Edward P. K. Parker

Corresponding author: edward.parker@lshtm.ac.uk

Contents:

P2: Supplementary Methods
P3: Supplementary Tables 1–7
P11: Supplementary Figures 1–7
P18: Citation details for studies included in meta-regression models
P21: Supplementary Methods references

Supplementary Methods

We adopted the following algorithm to transform effect sizes and confidence intervals (CIs) for meta-regression models, adapted from Wu et al[1], Cochrane guideline[2], and Harrer et al[3]:

1. All effect sizes and CIs were transformed to relative risks (RRs)
    a. When VE is 100, it was transformed to 99.5%
    b. When VE, upper CI, or lower CI is not between [-100;100], it was considered invalid and transformed to (NA)
    c. When the upper CI is 100, it was transformed to 99.9
    d. When the lower CI is 100, it was transformed to 97.5
    e. For VEs between [0;100]:
        i. RR= 1- (VE/100)
        ii. RR CI (95% CI = RR ± 1.96*SE):
            1. RR.CI.lower = 1 - (VE.CI.upper/100)
            2. RR.CI.upper = 1 - (VE.CI.lower/100)
    f. For VEs between [-100;0]:
        i. RR = 1/(1+VE/100)
        ii. RR CI (95% CI = RR±1.96*SE):
            1. RR.CI.lower = 1/(1 + VE.CI.upper/100)
            2. RR.CI.upper = 1/(1 + VE.CI.lower/100)
2. Effect estimates and CIs were transformed to a log-RR scale
        i. Intervention effect estimate = lnRR
        ii. SE_{logRR} = (ln(RR.CI.lower) - ln(RR.CI.upper))/3.92

CI, confidence interval; RR, relative risk; SE, standard error; VE, vaccine effectiveness

All analysis code is available on Github (https://github.com/marrpesce/COVID-global-vaccine-disparities).

Supplementary Table 1. Vaccine introduction and scale-up milestones by income status.

Milestone	Metric	GNI Q1 (lower income)	GNI Q2	GNI Q3	GNI Q4 (higher income)
Vaccine introduction	n/N (%) achieving milestone by 31 December 2021	50/51 (98%)	51/51 (100%)	51/51 (100%)	51/51 (100%)
	n/N (%) achieving milestone by 07 January 2024[a]	50/51 (98%)	51/51 (100%)	51/51 (100%)	51/51 (100%)
	Date achieved, median (IQR)	15/03/2021 (05/03/2021–05/04/2021)	24/02/2021 (31/01/2021–18/03/2021)	26/01/2021 (26/12/2020–19/02/2021)	30/12/2020 (23/12/2020–28/01/2021)
40% primary vaccine coverage	n/N (%) achieving milestone by 31 December 2021[b]	3/50 (6%)	20/51 (39%)	39/51 (77%)	47/51 (92%)
	Time in days since vaccine introduction, median (IQR) by 31 December 2021[b]	301 (242–322)	254 (215–274)	232 (181–262)	188 (160–208)
	Date achieved, median (IQR) by 31 December 2021[b]	24/12/2021 (18/10/2021–26/12/2021)	17/11/2021 (02/09/2021–30/11/2021)	01/09/2021 (18/07/2021–04/10/2021)	09/07/2021 (19/06/2021–09/08/2021)
	n/N (%) achieving milestone by 07 January 2024[a]	22/50 (44%)	31/51 (60.8%)	43/51 (84.3%)	49/51 (96.1%)
	Time in days since vaccine introduction, median (IQR) by 07 January 2024[a]	450 (347–637)	272 (224–356)	239 (182–274)	190 (160–209)
	Date achieved, median (IQR) by 07 January 2024[a]	29/06/2022 (17/02/2022–12/12/2022)	02/12/2021 (23/10/2021–24/01/2022)	10/09/2021 (23/07/2021–22/10/2021)	10/07/2021 (22/06/2021–12/08/2021)
70% primary vaccine coverage	n/N (%) achieving milestone by 30 June 2022[b]	2/50 (4%)	4/51 (8%)	16/51 (31%)	35/51 (69%)
	Time in days since vaccine introduction, median (IQR) by 30 June 2022[b]	350 (297–404)	326 (288–351)	376 (280–396)	263 (240–341)
	Date achieved, median (IQR) by 30 June 2022[b]	06/02/2022 (09/12/2021–05/04/2022)	05/01/2022 (13/12/2021–09/02/2022)	05/01/2022 (24/10/2021–07/02/2022)	19/10/2021 (28/08/2021–06/12/2021)
	n/N (%) achieving milestone by 07 January 2024[a]	5/50 (10%)	7/51 (14%)	19/51 (37%)	36/51 (71%)
	Time in days since vaccine introduction, median (IQR) by 07 January 2024[a]	579 (457–619)	379 (326–501)	384 (306–418)	268 (240–348)
	Date achieved, median (IQR) by 07 January 2024[a]	29/08/2022 (03/06/2022–11/12/2022)	02/05/2022 (05/01/2022–20/07/2022)	17/01/2022 (01/11/2021–09/03/2022)	22/10/2021 (29/08/2021–12/12/2021)

Data obtained from WHO and Our World in Data. GNI Q, per capita Gross National Income quartile; IQR, interquartile range. [a] Date of data extraction. [b] The milestones of 40% primary series coverage by the end of 2021 and 70% primary series coverage by mid-2022 align with the WHO global COVID-19 vaccination strategy.

Supplementary Table 2. Vaccine coverage in high-risk populations and overall according to WHO/UNICEF database.

Milestone	Period	Metric	GNI Q1 (lower income)	GNI Q2	GNI Q3	GNI Q4 (higher income)
N with vaccine introduction by 07 January 2024			50	51	51	51
Healthcare workers	December 2021	N countries/territories reporting	35	34	30	23
		% of total population in high-risk population (IQR)	0.4% (0.3–0.7%)	1% (0.7–1.5%)	2.1% (1.8–2.7%)	5.4% (3.4–6.9%)
		median coverage (IQR)	59% (31–100%)	97% (58–100%)	79% (50–95%)	76% (66–96%)
	December 2023	N countries/territories reporting	45	39	35	23
		% of total population in high-risk population (IQR)	0.4% (0.2–0.5%)	0.9% (0.7–1.3%)	2.1% (1.7–2.5%)	4.5% (3.2–6.5%)
		median coverage (IQR)	88% (56–100%)	100% (66–100%)	94% (60–100%)	83% (68–98%)
Older adults	December 2021	N countries/territories reporting	30	30	32	35
		% of total population in high-risk population (IQR)	9% (8–11%)	10% (8–17%)	16% (13–23%)	25% (17–27%)
		median coverage (IQR)	10% (4–27%)	50% (32–72%)	71% (63–85%)	89% (75–96%)
	December 2023	N countries/territories reporting	43	41	38	37
		% of total population in high-risk population (IQR)	9% (8–11%)	10% (8–12%)	17% (12–22%)	26% (19–27%)
		median coverage (IQR)	47% (18–90%)	72% (43–86%)	83% (70–98%)	93% (81–99%)
Total population	December 2021	N countries/territories reporting	50	51	51	45
		median coverage (IQR)	8% (3–21%)	39% (23–48%)	55% (40–67%)	70% (64–75%)
	December 2023	N countries/territories reporting	50	51	51	45
		median coverage (IQR)	38% (22–53%)	50% (36–68%)	64% (51–81%)	78% (72–83%)

Data obtained from WHO/UNICEF. Vaccination data collection methods and denominator inclusion criteria for healthcare workers were not defined in publicly available data and may have varied across countries and territories. The age thresholds to define older adults are provided in Supplementary Table 3. GNI Q, per capita Gross National Income quartile; IQR, interquartile range.

Supplementary Table 3. Age thresholds for defining older adults.

Year	GNI quartile	N countries/ territories	N reporting	Age threshold (years)						
				45+	50+	55+	60+	65+	75+	Not defined
2021	1 (lower income)	51	30	0	13	4	10	3	0	0
	2	51	30	1	3	2	13	2	0	9
	3	51	32	0	0	0	7	1	0	24
	4 (higher income)	51	35	0	0	0	5	5	1	24
2023	1 (lower income)	51	43	1	25	2	8	3	0	4
	2	51	41	1	2	0	7	0	0	31
	3	51	38	0	1	0	3	0	0	34
	4 (higher income)	51	37	0	0	0	0	3	0	34

GNI, per capita Gross National Income.

Supplementary Table 4. Selection of comparisons eligible for meta-regression.

Vaccine	Variant	Outcome	N studies by GNI quartile Q1	Q2	Q3	Q4	Sufficient studies (≥10)	Sufficient studies outside majority quartile (≥2)	Eligible	Eligible for sensitivity analysis restricted to >60s	Eligible for sensitivity analysis restricted to <26 week follow-up
BioNTech/Pfizer BNT162b2	Alpha	Death	0	0	1	5	No	No	No	–	–
		Severe	0	0	0	12	Yes	No	No	–	–
	Delta	Death	0	0	4	4	No	Yes	No	–	–
		Severe	0	1	6	19	Yes	Yes	Yes	No	Yes
	Omicron	Death	0	0	0	5	No	No	No	–	–
		Severe	0	1	5	18	Yes	Yes	Yes	No	Yes
Janssen Ad26.COV2.S	Alpha	Death	0	0	0	0	No	No	No	–	–
		Severe	0	0	0	2	No	No	No	–	–
	Delta	Death	0	0	2	1	No	No	No	–	–
		Severe	0	0	4	5	No	Yes	No	–	–
	Omicron	Death	0	0	0	0	No	No	No	–	–
		Severe	0	0	2	1	No	No	No	–	–
Moderna mRNA-1273	Alpha	Death	0	0	1	1	No	No	No	–	–
		Severe	0	0	0	3	No	No	No	–	–
	Delta	Death	0	0	2	2	No	Yes	No	–	–
		Severe	0	1	3	15	Yes	Yes	Yes	No	No
	Omicron	Death	0	0	0	2	No	No	No	–	–
		Severe	0	1	2	10	Yes	Yes	Yes	No	No
Oxford/ AstraZeneca ChAdOx1-S	Alpha	Death	0	0	1	1	No	No	No	–	–
		Severe	0	0	0	3	No	No	No	–	–
	Delta	Death	0	1	3	2	No	Yes	No	–	–
		Severe	2	2	6	8	Yes	Yes	Yes	No	Yes
	Omicron	Death	0	0	0	1	No	No	No	–	–
		Severe	0	2	3	5	Yes	Yes	Yes	No	No
Sinovac CoronaVac	Alpha	Death	0	0	0	0	No	No	No	–	–
		Severe	0	0	0	0	No	No	No	–	–
	Delta	Death	0	0	1	0	No	No	No	–	–
		Severe	0	0	3	0	No	No	No	–	–
	Omicron	Death	0	0	1	3	No	No	No	–	–
		Severe	0	0	3	3	No	Yes	No	–	–

GNI, per capita Gross National Income; Q, quartile.

Supplementary Table 5. Characteristics of primary series vaccine effectiveness studies included in meta-regression.

		Total (N = 51)	BNT162b2 (N = 42)	mRNA-1273 (N = 26)	ChAdOx1-S (N = 22)
Vaccines[a]	BNT162b2	42 (82%)	–	–	–
	mRNA-1273	26 (51%)	–	–	–
	ChAdOx1-S	22 (43%)	–	–	–
Variant[a]	Delta	34 (67%)	26 (62%)	19 (73%)	18 (82%)
	Omicron	28 (55%)	24 (57%)	13 (50%)	10 (45%)
GNI quartile	1 (lower income)	2 (4%)	0 (0%)	0 (0%)	2 (9%)
	2	2 (4%)	1 (2%)	1 (4%)	2 (9%)
	3	9 (18%)	8 (19%)	4 (15%)	7 (32%)
	4 (higher income)	38 (75%)	33 (79%)	21 (81%)	11 (50%)

Data are n studies (%). GNI, per capita Gross National Income. [a] Individual studies could report on multiple groups; percentages may therefore sum to more than 100%.

Supplementary Table 6. Meta-regression of primary series vaccine effectiveness by income status.

Vaccine	Variant	Metric	GNI Q1	GNI Q2	GNI Q3	GNI Q4	Moderator test (p-value)	I^2, level 2	I^2, level 3	τ, level 2	τ, level 3
BNT162b2	Delta	RR (95% CI)	–	0.08 (0.01–0.71)	0.09 (0.05–0.19)	0.06 (0.04–0.11)	0.7074	43.31	54.80	0.57	0.64
		95% PI for RR	–	0.004–1.32	0.01–0.60	0.01–0.40					
		VE (95% CI)	–	92% (29–99)	91% (81–95)	94% (89–96)					
		N countries/territories	–	1	6	9					
		N studies	–	1	6	19					
		N estimates	–	1	8	20					
		Follow-up start, median (IQR) weeks	–	2 (NA)	2 (2–2)	2 (2–2)					
		Follow-up limit, median (IQR) weeks	–	38 (NA)	28 (16–48)	25 (21–35)					
	Omicron	RR (95% CI)	–	0.51 (0.13–1.96)	0.33 (0.18–0.62)	0.26 (0.16–0.43)	0.6184	17.59	79.07	0.25	0.53
		95% PI for RR	–	0.08–3.13	0.08–1.29	0.07–0.98					
		VE (95% CI)	–	49% (-49–87)	67% (38–82)	74% (57–84)					
		N countries/territories	–	1	4	6					
		N studies	–	1	5	18					
		N estimates	–	1	5	23					
		Follow-up start, median (IQR) weeks	–	2 (NA)	2 (2–2)	2 (2–2)					
		Follow-up limit, median (IQR) weeks	–	53 (NA)	11 (8–24)	42 (24–52)					
mRNA-1273	Delta	RR (95% CI)	–	0.24 (0.01–6.95)	0.03 (0.01–0.13)	0.04 (0.02–0.10)	0.4829	65.94	31.17	0.90	0.62
		95% PI for RR	–	0.00–14.33	0.00–0.45	0.00–0.52					
		VE (95% CI)	–	76% (-86–99)	97% (87–99)	96% (90–98)					
		N countries/territories	–	1	2	6					
		N studies[a]	–	1	2	14					
		N estimates	–	1	4	14					
		Follow-up start, median (IQR) weeks	–	2 (NA)	2 (2–2)	2 (2–2)					
		Follow-up limit, median (IQR) weeks	–	37 (NA)	48 (40–48)	25 (22–35)					

Vaccine	Variant	Metric	GNI Q1	GNI Q2	GNI Q3	GNI Q4	Moderator test (p-value)	I^2, level 2	I^2, level 3	τ, level 2	τ, level 3
mRNA-1273	Omicron	RR (95% CI)	–	0.58 (0.09–3.73)	0.25 (0.07–0.96)	0.34 (0.18–0.64)	0.7314	61.04	31.31	0.43	0.31
		95% PI for RR	–	0.06–5.25	0.04–1.50	0.09–1.30					
		VE (95% CI)	–	42% (-73–91)	75% (4–93)	66% (36–82)					
		N countries/territories	–	1	2	3					
		N studies	–	1	2	10					
		N estimates	–	1	2	10					
		Follow-up start, median (IQR) weeks	–	2 (NA)	2 (2–2)	2 (2–2)					
		Follow-up limit, median (IQR) weeks	–	52 (NA)	18 (14–21)	47 (42–56)					
ChAdOx1-S	Delta	RR (95% CI)	0.11 (0.01–0.88)	0.17 (0.02–1.25)	0.11 (0.04–0.28)	0.09 (0.03–0.26)	0.9573	28.89	69.44	0.53	0.83
		95% PI for RR	0.01–2.10	0.01–3.03	0.01–1.09	0.01–0.96					
		VE (95% CI)	89% (12–99)	83% (-20–98)	89% (72–96)	91% (74–97)					
		N countries/territories	1	1	6	4					
		N studies	2	2	6	8					
		N estimates	2	2	8	8					
		Follow-up start, median (IQR) weeks	2 (1–2)	2 (2–2)	2 (2–2)	2 (2–5)					
		Follow-up limit, median (IQR) weeks	18 (15–20)	35 (34–36)	26 (16–48)	21 (16–29)					
	Omicron	RR (95% CI)	–	0.90 (0.33–2.44)	0.32 (0.14–0.73)	0.34 (0.18–0.65)	0.1821	<0.001	88.63	<0.001	0.39
		95% PI for RR	–	0.23–3.52	0.09–1.11	0.11–1.06					
		VE (95% CI)	–	10% (-59–67)	68% (27–86)	66% (35–82)					
		N countries/territories	–	1	2	3					
		N studies[b]	–	2	2	5					
		N estimates	–	2	2	6					
		Follow-up start, median (IQR) weeks	–	2 (2–2)	2 (2–2)	5 (2–12)					
		Follow-up limit, median (IQR) weeks	–	50 (49–51)	26 (17–34)	25 (25–40)					

Three-level meta-regression models were fitted with GNI quartile as an explanatory variable for the moderator test. Country/territory was included as a random effect to account for the anticipated clustering of estimates. I^2 quantifies the proportion of variability within countries/territories (level 2) and between countries/territories (level 3). τ is an estimate of the standard deviation of effect sizes, presented within countries/territories (level 2) and between countries/territories (level 3). [a] 2 studies (2 VE estimates) excluded during model fitting as log RR or its standard error could not be derived. [b] 1 study (1 VE estimate) excluded during model fitting as log RR or its standard error could not be derived. GNI Q, per capita Gross National Income quartile; IQR, interquartile range; NA, not applicable; PI, prediction interval; RR, relative risk; VE, vaccine effectiveness.

Supplementary Table 7. Sensitivity analysis of primary series vaccine effectiveness meta-regression with maximum upper follow-up limit of 26 weeks.

Vaccine	Variant	Metric	GNI Q1	GNI Q3	GNI Q4	Moderator test (p-value)	I^2, level 2	I^2, level 3	τ, level 2	τ, level 3
BNT162b2	Delta	RR (95% CI)	–	0.13 (0.07–0.26)	0.07 (0.04–0.11)	0.1070	26.88	69.24	0.33	0.53
		95% PI for RR	–	0.03–0.60	0.02–0.28					
		VE (95% CI)	–	87% (74–93)	93% (89–96)					
		N countries/territories	–	4	8					
		N studies	–	4	10					
		N estimates	–	4	11					
		Follow-up start, median (IQR) weeks	–	2 (2–2)	2 (2–2)					
		Follow-up limit, median (IQR) weeks	–	15 (12–18)	21 (16–23)					
	Omicron	RR (95% CI)	–	0.29 (0.14–0.58)	0.26 (0.16–0.44)	0.8089	14.14	82.12	0.22	0.52
		95% PI for RR	–	0.07–1.17	0.07–0.97					
		VE (95% CI)	–	71% (42–86)	74% (56–84)					
		N countries/territories	–	3	6					
		N studies	–	4	8					
		N estimates	–	4	11					
		Follow-up start, median (IQR) weeks	–	2 (2–2)	2 (2–2)					
		Follow-up limit, median (IQR) weeks	–	10 (7–14)	24 (11–25)					
ChAdOx1-S	Delta	RR (95% CI)	0.18 (0.08–0.40)	0.10 (0.05–0.19)	0.10 (0.03–0.37)	0.4697	95.95	<0.001	0.61	<0.001
		95% PI for RR	0.04–0.89	0.02–0.46	0.02–0.68					
		VE (95% CI)	82% (60–92)	90% (81–95)	90% (63–97)					
		N countries/territories	1	4	4					
		N studies	2	4	6					
		N estimates	2	4	6					
		Follow-up start, median (IQR) weeks	2 (1–2)	2 (2–6)	2 (2–2)					
		Follow-up limit, median (IQR) weeks	18 (15–20)	15 (13–18)	20 (8–21)					

Three-level meta-regression models were fitted with GNI quartile as an explanatory variable for the moderator test. Country/territory was included as a random effect to account for the anticipated clustering of estimates. I^2 quantifies the proportion of variability within countries/territories (level 2) and between countries/territories (level 3). τ is an estimate of the standard deviation of effect sizes, presented within countries/territories (level 2) and between countries/territories (level 3). GNI Q, per capita Gross National Income quartile; IQR, interquartile range; NA, not applicable; PI, prediction interval; RR, relative risk; VE, vaccine effectiveness.

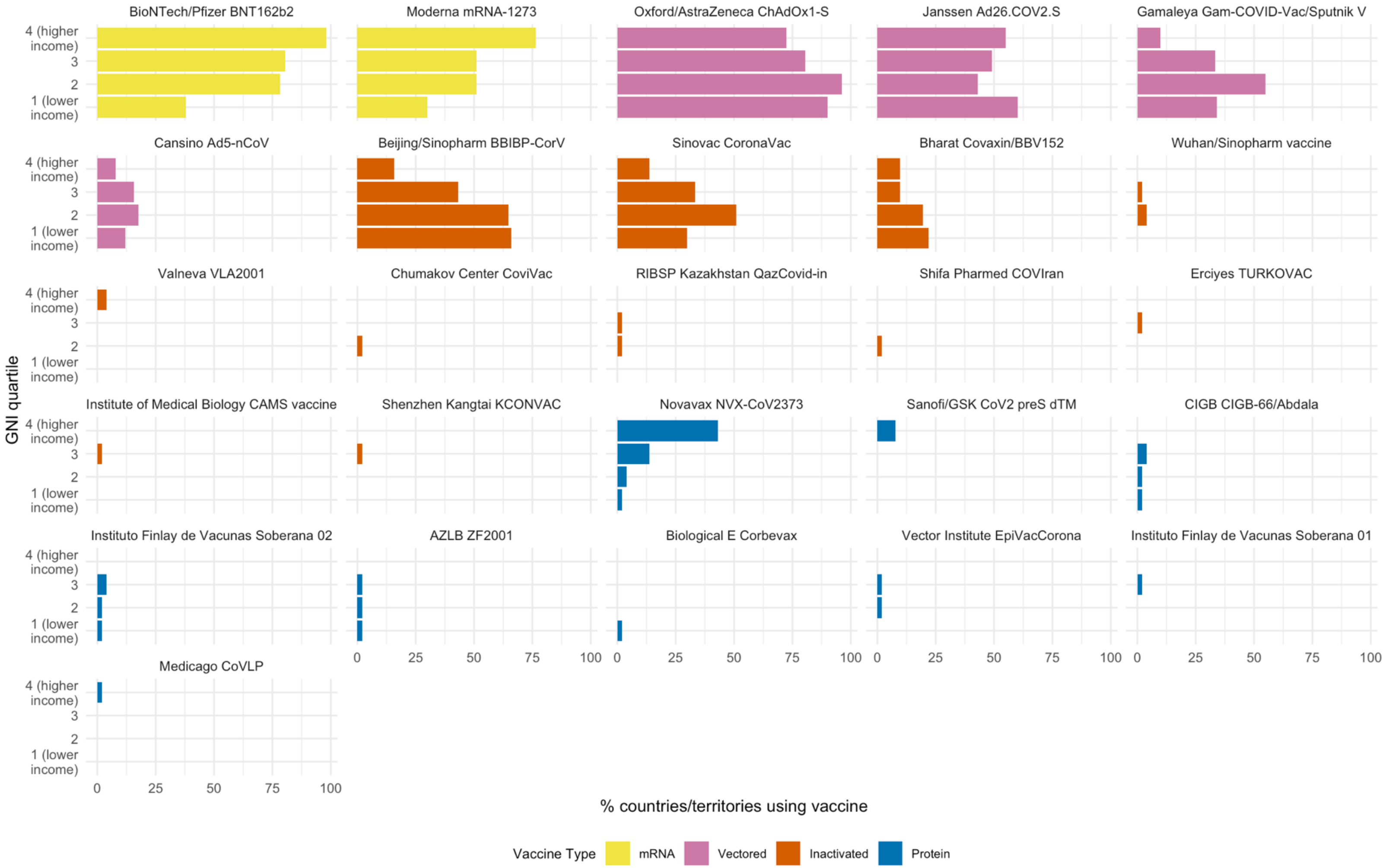

Supplementary Fig. 1. Percentage of countries and territories using different vaccine products by income status. Data reflect any use of the specified product in a country or territory, as listed by Our World in Data on 07 January 2024. The breakdown of doses by vaccine product is not systematically reported in available data. N countries/territories = 51, 51, 51, and 50 for quartiles 4, 3, 2, and 1, respectively. AZLB, Anhui Zhifei Longcom Biopharmaceutical; GNI, per capita Gross National Income; RIBSP, Research Institute for Biological Safety Problems.

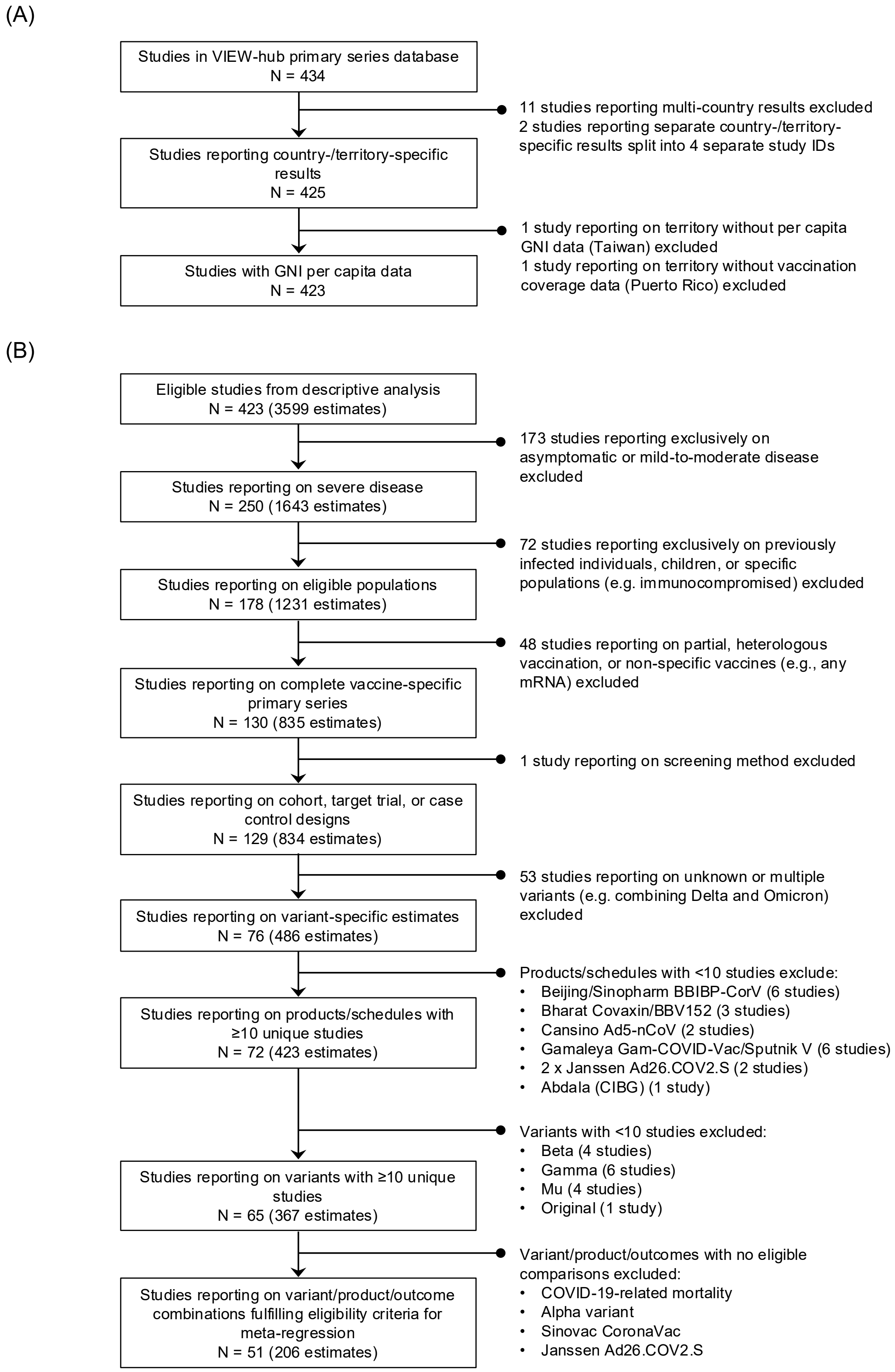

Supplementary Fig. 2. Flow chart of vaccine effectiveness study selection. (A) Selection of studies for descriptive analyses. The VIEW-hub primary series database was obtained on 11 January 2024. (B) Selection of studies for meta-regression. See Supplementary Table 4 for assessment of eligibility criteria for meta-regression.

(A)

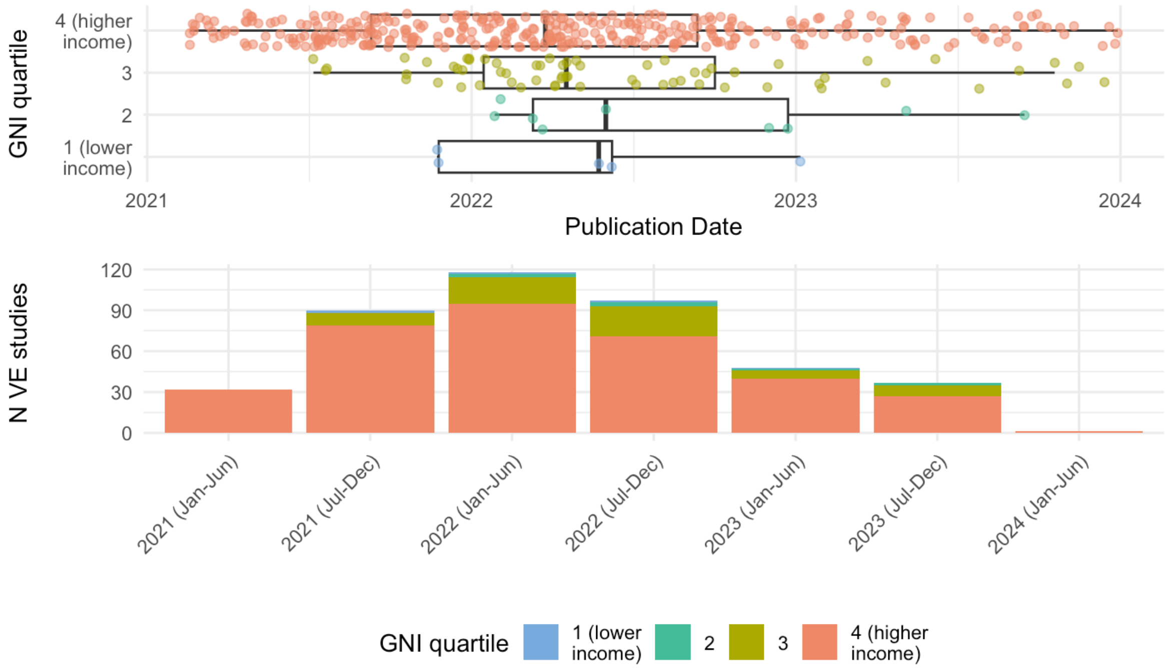

(B)

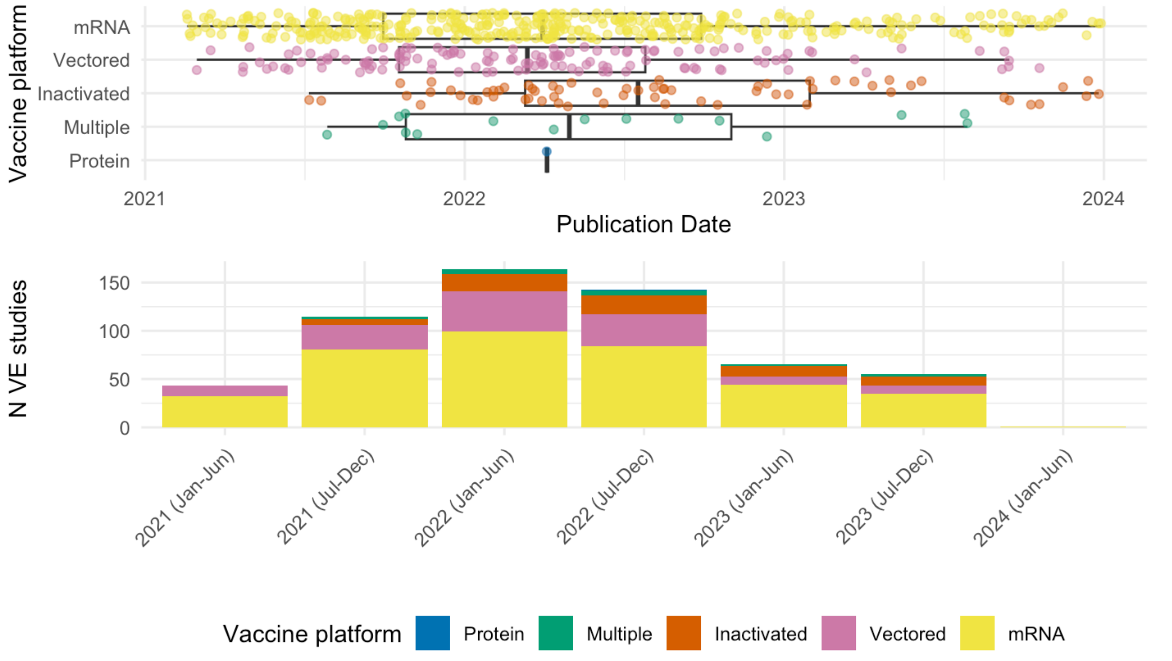

Supplementary Fig. 3. Distribution of vaccine effectiveness papers over time. Data are displayed by (A) GNI quartile (n publications = 345, 64, 9, and 5 for quartiles 4, 3, 2, and 1, respectively) and (B) vaccine platform (n publications = 376 for mRNA, 128 for vectored, 65 for inactivated, 16 for multiple, and 1 for protein). In the upper panels, each point represents a study. VE data were obtained on 11 January 2024 from VIEW-hub. Where peer-reviewed publications were preceded by preprints, we assigned estimates to the earliest date available. GNI, per capita Gross National Income; VE, vaccine effectiveness.

BNT162b2 – Delta

Study	Country/territory	Population	Max. follow-up (w)	RR (95% CI)
**GNI quartile 2**				
Penayo (2022)	Paraguay	general pop	38	0.08 (0.02-0.28)
**Summary**				**0.08 (0.01-0.71)**
**GNI quartile 3**				
Paternina-Caicedo (2023)	Colombia	general pop	36	0.09 (0.04-0.21)
Smid (2022)	Czech Republic	general pop	11	0.20 (0.15-0.28)
Lytras (2022)	Greece	15-59 years	48	0.02 (0.01-0.03)
Lytras (2022)	Greece	60-79 years	48	0.03 (0.03-0.04)
Lytras (2022)	Greece	>= 80 years	48	0.06 (0.04-0.08)
Voko (2022)	Hungary	18-64 years	17	0.17 (0.15-0.20)
Hernandez-Avila (2023)	Mexico	>=65 years	13	0.12 (0.10-0.15)
Collie (2021)	South Africa	general pop	19	0.07 (0.06-0.10)
**Summary**				**0.09 (0.05-0.19)**
**GNI quartile 4**				
Nasreen (2022)	Canada	general pop	28	0.02 (0.01-0.04)
Skowronski (2022)	Canada	general pop	38	0.02 (0.02-0.03)
Baum (2022)	Finland	>= 70 years	13	0.10 (0.04-0.22)
Golberg (2021)	Israel	40-59 years	23	0.08 (0.05-0.13)
Golberg (2021)	Israel	>= 60 years	23	0.15 (0.12-0.19)
Mimura (2023)	Japan	>=65 years	22	0.06 (0.00-0.16)
Starrfelt (2022)	Norway	general pop	9	0.03 (0.01-0.04)
Tang (2021)	Qatar	general pop	25	0.06 (0.02-0.14)
Altarawneh (2023)	Qatar	general pop	48	0.09 (0.06-0.14)
Monge (2022)	Spain	50-59 years	21	0.03 (0.02-0.03)
Stowe (2021)	UK	general pop	20	0.04 (0.01-0.14)
Andrews (2022)	UK	general pop	34	0.03 (0.03-0.04)
Chatzilena (2022)	UK	general pop	56	0.17 (0.13-0.24)
Tartof (2021)	USA	general pop	29	0.07 (0.04-0.16)
Grannis (2021)	USA	general pop	4	0.20 (0.15-0.27)
Tartof (2022)	USA	general pop	47	0.24 (0.18-0.31)
Lauring (2022)	USA	general pop	27	0.18 (0.16-0.20)
Plumb (2022)	USA	general pop	55	0.50 (0.41-0.61)
Lin (2022)	USA	general pop	25	0.14 (0.12-0.17)
Embi (2023)	USA	non-ICP adults	21	0.10 (0.09-0.12)
**Summary**				**0.06 (0.04-0.11)**

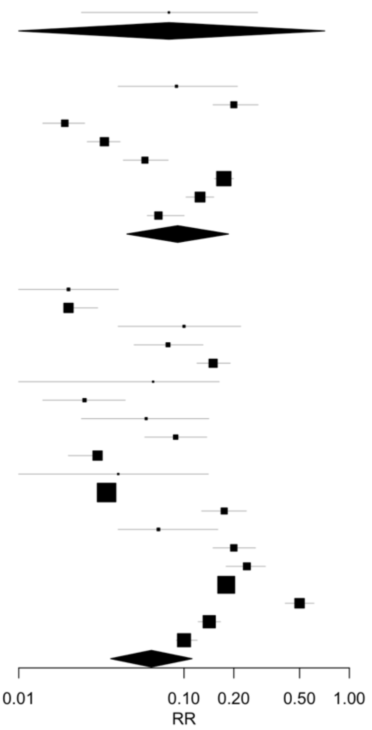

BNT162b2 – Omicron

Study	Country/territory	Population	Max. follow-up (w)	RR (95% CI)
**GNI quartile 2**				
Penayo (2022)	Paraguay	general pop	53	0.50 (0.28-0.90)
**Summary**				**0.51 (0.13-1.96)**
**GNI quartile 3**				
Cerqueira-Silva (2023)	Brazil	general pop	8	0.17 (0.15-0.19)
Paternina-Caicedo (2023)	Colombia	general pop	47	0.55 (0.30-0.99)
Smid (2022)	Czech Republic	general pop	11	0.54 (0.40-0.72)
Collie (2021)	South Africa	general pop	24	0.30 (0.24-0.38)
Collie (2022)	South Africa	general pop	4	0.20 (0.10-0.37)
**Summary**				**0.33 (0.18-0.62)**
**GNI quartile 4**				
Hansen (2022)	Denmark	general pop	4	0.50 (0.37-0.66)
Baum (2022)	Finland	>= 70 years	13	0.09 (0.05-0.17)
Yan (2022)	Hong Kong SAR	18-50 years	52	0.26 (0.15-0.46)
Yan (2022)	Hong Kong SAR	51-64 years	52	0.16 (0.09-0.28)
Yan (2022)	Hong Kong SAR	>= 65 years	52	0.18 (0.13-0.26)
Wan (2022)	Hong Kong SAR	60-79 years	24	0.25 (0.23-0.28)
Wan (2022)	Hong Kong SAR	>=80 years	24	0.19 (0.16-0.22)
Yan (2023)	Hong Kong SAR	general pop	4	0.27 (0.24-0.31)
Chemaitelly (2022)	Qatar	general pop	26	0.30 (0.15-0.55)
Altarawneh (2022)	Qatar	general pop	44	0.26 (0.18-0.40)
Stowe (2022)	UK	18-64 years	25	0.26 (0.18-0.38)
Stowe (2022)	UK	>= 65 years	25	0.12 (0.07-0.21)
Cerqueira-Silva (2023)	UK	general pop	8	0.10 (0.01-0.70)
Tartof (2022)	USA	general pop	47	0.38 (0.31-0.47)
Plumb (2022)	USA	general pop	55	0.63 (0.53-0.74)
Sharma (2022)	USA	veterans	42	0.47 (0.42-0.52)
Adams (2022)	USA	general pop	72	0.64 (0.52-0.79)
Tartof (2022)	USA	general pop	26	0.46 (0.35-0.62)
Tartof (2022)	USA	general pop	26	0.44 (0.19-1.02)
Risk (2022)	USA	general pop	56	0.33 (0.22-0.49)
Link-Gelles (2022)	USA	general pop	82	0.83 (0.73-0.93)
Lin (2022)	USA	general pop	47	0.41 (0.34-0.51)
Tartof (2022)	USA	general pop	84	1.04 (0.50- NA)
**Summary**				**0.26 (0.16-0.43)**

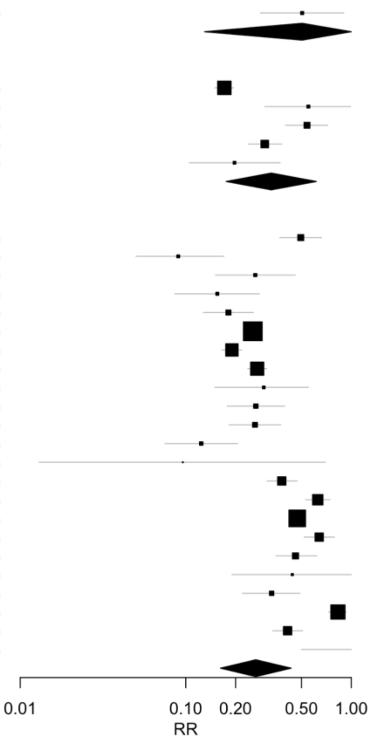

Supplementary Fig. 4. Forest plot for meta-regression of BNT162b2 primary series vaccine effectiveness against severe COVID-19. RRs reflect estimated protection in vaccinated versus unvaccinated individuals. Summary estimates are derived from three-level meta-regression models (see Supplementary Table 6). Axis limits and CIs are truncated at 0.01 and 1.0 for visualisation purposes. CI, confidence interval; GNI, per capita Gross National Income; RR, relative risk; w, weeks.

mRNA-1273 – Delta

Study	Country/territory	Population	Max. follow-up (w)	RR (95% CI)
**GNI quartile 2**				
Penayo (2022)	Paraguay	general pop	37	0.24 (0.03- NA)
**Summary**				**0.24 (0.01-6.95)**
**GNI quartile 3**				
Lytras (2022)	Greece	15-59 years	48	0.01 (0.00-0.02)
Lytras (2022)	Greece	60-79 years	48	0.01 (0.00-0.03)
Lytras (2022)	Greece	>= 80 years	48	0.02 (0.00-0.10)
Voko (2022)	Hungary	18-64 years	17	0.15 (0.09-0.25)
**Summary**				**0.03 (0.01-0.13)**
**GNI quartile 4**				
Nasreen (2022)	Canada	general pop	25	0.02 (0.00-0.07)
Skowronski (2022)	Canada	general pop	38	0.03 (0.02-0.03)
Baum (2022)	Finland	>= 70 years	13	0.08 (0.01-0.58)
Starrfelt (2022)	Norway	general pop	25	0.05 (0.03-0.09)
Tang (2021)	Qatar	general pop	25	0.04 (0.00-0.29)
Altarawneh (2023)	Qatar	general pop	48	0.02 (0.00-0.09)
Monge (2022)	Spain	50-59 years	21	0.02 (0.01-0.03)
Grannis (2021)	USA	general pop	4	0.05 (0.03-0.08)
Bruxvoort (2021)	USA	general pop	25	0.03 (0.01-0.07)
Tseng (2022)	USA	general pop	48	0.01 (0.00-0.07)
Lauring (2022)	USA	general pop	27	0.12 (0.10-0.14)
Plumb (2022)	USA	general pop	55	0.56 (0.45-0.70)
Lin (2022)	USA	general pop	24	0.10 (0.09-0.12)
Embi (2023)	USA	non-ICP adults	21	0.05 (0.05-0.08)
**Summary**				**0.04 (0.02-0.10)**

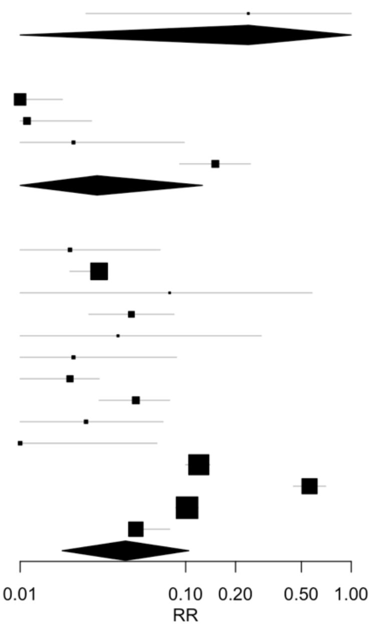

mRNA-1273 – Omicron

Study	Country/territory	Population	Max. follow-up (w)	RR (95% CI)
**GNI quartile 2**				
Penayo (2022)	Paraguay	general pop	52	0.58 (0.16- NA)
**Summary**				**0.58 (0.09-3.73)**
**GNI quartile 3**				
Paternina-Caicedo (2023)	Colombia	general pop	25	0.05 (0.01-0.46)
Smid (2022)	Czech Republic	general pop	11	0.49 (0.20-1.25)
**Summary**				**0.25 (0.07-0.96)**
**GNI quartile 4**				
Baum (2022)	Finland	>= 70 years	13	0.21 (0.08-0.57)
Chemaitelly (2022)	Qatar	general pop	26	0.13 (0.03-0.60)
Altarawneh (2022)	Qatar	general pop	44	0.34 (0.18-0.62)
Tseng (2022)	USA	general pop	48	0.16 (0.03-0.77)
Plumb (2022)	USA	general pop	55	0.64 (0.53-0.78)
Sharma (2022)	USA	veterans	42	0.33 (0.28-0.39)
Adams (2022)	USA	general pop	72	0.59 (0.46-0.75)
Risk (2022)	USA	general pop	56	0.21 (0.12-0.37)
Link-Gelles (2022)	USA	general pop	82	0.72 (0.62-0.83)
Lin (2022)	USA	general pop	46	0.35 (0.29-0.43)
**Summary**				**0.34 (0.18-0.64)**

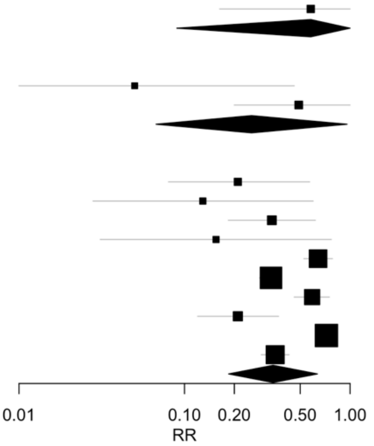

Supplementary Fig. 5. Forest plot for meta-regression of mRNA-1273 primary series vaccine effectiveness against severe COVID-19. RRs reflect estimated protection in vaccinated versus unvaccinated individuals. Summary estimates are derived from three-level meta-regression models (see Supplementary Table 6). Axis limits and CIs are truncated at 0.01 and 1.0 for visualisation purposes. CI, confidence interval; GNI, per capita Gross National Income; RR, relative risk; w, weeks.

ChAdOx1-S – Delta

Study	Country/territory	Population	Max. follow-up (w)	RR (95% CI)
**GNI quartile 1**				
Tsundue (2022)	India	general pop	13	0.01 (0.00-0.10)
Battacharya (2023)	India	general pop	22	0.21 (0.19-0.47)
**Summary**				**0.11 (0.01-0.88)**
**GNI quartile 2**				
Penayo (2022)	Paraguay	general pop	33	0.17 (0.09-0.34)
Irala (2023)	Paraguay	general pop	37	0.17 (0.08-0.32)
**Summary**				**0.17 (0.02-1.25)**
**GNI quartile 3**				
Clemens (2022)	Brazil	18-60 years	12	0.00 (0.00-0.56)
Paternina-Caicedo (2023)	Colombia	general pop	32	0.29 (0.11-0.73)
Smid (2022)	Czech Republic	general pop	19	0.20 (0.11-0.38)
Lytras (2022)	Greece	15-59 years	48	0.02 (0.01-0.05)
Lytras (2022)	Greece	60-79 years	48	0.03 (0.02-0.05)
Lytras (2022)	Greece	>= 80 years	48	0.02 (0.01-0.08)
Voko (2022)	Hungary	18-64 years	17	0.24 (0.19-0.29)
Hernandez-Avila (2023)	Mexico	>=65 years	13	0.19 (0.16-0.22)
**Summary**				**0.11 (0.04-0.28)**
**GNI quartile 4**				
Nasreen (2022)	Canada	general pop	3	0.10 (0.03-0.33)
Skowronski (2022)	Canada	general pop	38	0.05 (0.03-0.07)
Baum (2022)	Finland	>= 70 years	26	0.21 (0.13-0.33)
Monge (2022)	Spain	50-59 years	21	0.04 (0.02-0.07)
Stowe (2021)	UK	general pop	20	0.08 (0.03-0.25)
Andrews (2022)	UK	general pop	20	0.07 (0.07-0.08)
Katikireddi (2021)	UK	general pop	4	0.16 (0.13-0.20)
Kirsebom (2022)	UK	>= 65 years	44	0.27 (0.24-0.30)
**Summary**				**0.09 (0.03-0.26)**

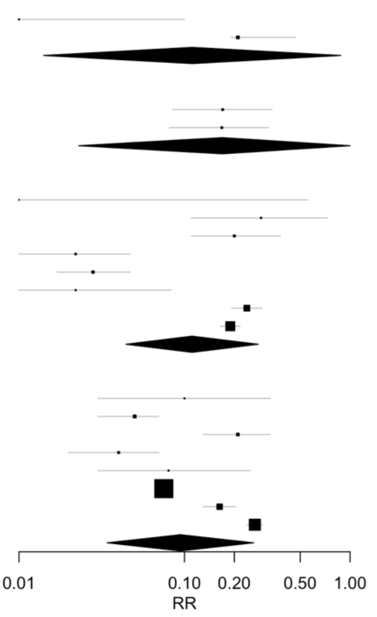

ChAdOx1-S – Omicron

Study	Country/territory	Population	Max. follow-up (w)	RR (95% CI)
**GNI quartile 2**				
Penayo (2022)	Paraguay	general pop	48	0.90 (0.62-1.45)
Irala (2023)	Paraguay	general pop	52	0.90 (0.62-1.46)
**Summary**				**0.90 (0.33-2.44)**
**GNI quartile 3**				
Cerqueira-Silva (2023)	Brazil	general pop	8	0.23 (0.19-0.27)
Paternina-Caicedo (2023)	Colombia	general pop	43	0.90 (0.45-4.55)
**Summary**				**0.32 (0.14-0.73)**
**GNI quartile 4**				
Baum (2022)	Finland	>= 70 years	26	0.63 (0.15- NA)
Park (2023)	South Korea	>=60 years	80	0.28 (0.26-0.30)
Stowe (2022)	UK	18-64 years	25	0.41 (0.25-0.68)
Stowe (2022)	UK	>= 65 years	25	0.29 (0.17-0.50)
Kirsebom (2022)	UK	>= 65 years	44	0.39 (0.30-0.50)
Cerqueira-Silva (2023)	UK	general pop	21	0.21 (0.07-0.68)
**Summary**				**0.34 (0.18-0.65)**

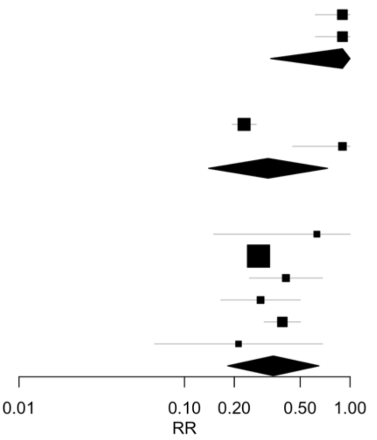

Supplementary Fig. 6. Forest plot for meta-regression of ChAdOx1-S primary series vaccine effectiveness against severe COVID-19. RRs reflect estimated protection in vaccinated versus unvaccinated individuals. Summary estimates are derived from three-level meta-regression models (see Supplementary Table 6). Axis limits and CIs are truncated at 0.01 and 1.0 for visualisation purposes. CI, confidence interval; GNI, per capita Gross National Income; RR, relative risk; w, weeks.

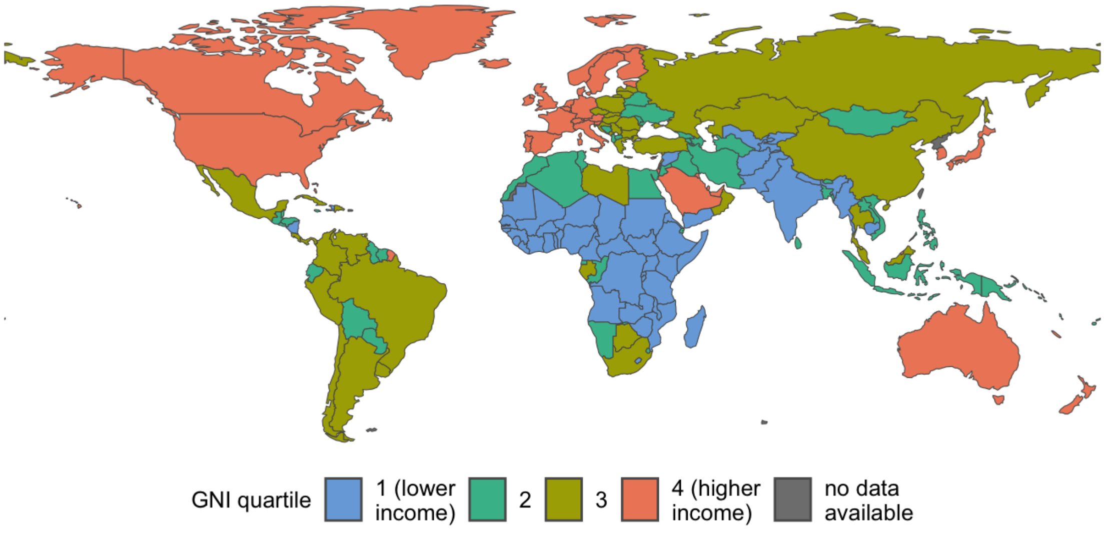

Supplementary Fig. 7. Map of countries and territories by per capita Gross National Income quartile. World Bank estimates of GNI per capita are based on the Atlas method. GNI per capita ranged from 220–2170, 2180–6520, 6570–22100, and 23060–116920 in quartiles 1 (lowest income), 2, 3, and 4 (highest income), respectively. Territories without GNI data included: American Samoa, Channel Islands, Gibraltar, Guam, Saint Martin (French part), Monaco, Northern Mariana Islands, Democratic People's Republic of Korea, British Virgin Islands, US Virgin Islands, and Taiwan. In addition, the Marshall Islands, the Federated States of Micronesia, and Puerto Rico were excluded from descriptive analyses as vaccine coverage metrics were not included in WHO or Our World in Data databases despite records of vaccine introductions in the public domain. GNI, per capita Gross National Income.

Citation details for studies included in meta-regression models

1. Adams, K. *et al.* Vaccine effectiveness of primary series and booster doses against covid-19 associated hospital admissions in the United States: living test negative design study. *BMJ* **379**, e072065 (2022).
2. Altarawneh, H. N. *et al.* Effects of Previous Infection and Vaccination on Symptomatic Omicron Infections. *N Engl J Med* **387**, 21–34 (2022).
3. Altarawneh, H. N. *et al.* Effects of previous infection, vaccination, and hybrid immunity against symptomatic Alpha, Beta, and Delta SARS-CoV-2 infections: an observational study. *eBioMedicine* **95**, (2023).
4. Andrews, N. *et al.* Duration of Protection against Mild and Severe Disease by Covid-19 Vaccines. *N Engl J Med* **386**, 340–350 (2022).
5. Baum, U. *et al.* High vaccine effectiveness against severe COVID-19 in the elderly in Finland before and after the emergence of Omicron. *BMC Infectious Diseases* **22**, 816 (2022).
6. Bhattacharya, D. *et al.* Effectiveness of the BBV-152 and AZD1222 vaccines among adult patients hospitalized in tertiary hospitals in Odisha with symptomatic respiratory diseases: A test-negative case–control study. *Front. Public Health* **10**, (2023).
7. Bruxvoort, K. J. *et al.* Effectiveness of mRNA-1273 against delta, mu, and other emerging variants of SARS-CoV-2: test negative case-control study. *BMJ* **375**, e068848 (2021).
8. Cerqueira-Silva, T. *et al.* Effectiveness of mRNA boosters after homologous primary series with BNT162b2 or ChAdOx1 against symptomatic infection and severe COVID-19 in Brazil and Scotland: A test-negative design case–control study. *PLOS Medicine* **20**, e1004156 (2023).
9. Chatzilena, A. *et al.* Effectiveness of BNT162b2 COVID-19 vaccination in prevention of hospitalisations and severe disease in adults with SARS-CoV-2 Delta (B.1.617.2) and Omicron (B.1.1.529) variant between June 2021 and July 2022: a prospective test negative case–control study. *The Lancet Regional Health - Europe* **25**, 100552 (2023).
10. Chemaitelly, H. *et al.* Duration of mRNA vaccine protection against SARS-CoV-2 Omicron BA.1 and BA.2 subvariants in Qatar. *Nat Commun* **13**, 3082 (2022).
11. Collie, S. *et al*. Effectiveness of BNT162b2 Vaccine against Omicron Variant in South Africa. *New England Journal of Medicine* **386**, 494–496 (2022).
12. Collie, S. *et al.* Effectiveness and Durability of the BNT162b2 Vaccine against Omicron Sublineages in South Africa. *N Engl J Med* **387**, 1332–1333 (2022).
13. Costa Clemens, S. A. *et al.* Effectiveness of the Fiocruz recombinant ChadOx1-nCoV19 against variants of SARS-CoV-2 in the Municipality of Botucatu-SP. *Front. Public Health* **10**, (2022).
14. Embi, P. J. *et al.* Effectiveness of COVID-19 vaccines at preventing emergency department or urgent care encounters and hospitalizations among immunocompromised adults: An observational study of real-world data across 10 US states from August-December 2021. *Vaccine* **41**, 5424–5434 (2023).
15. Goldberg Yair *et al.* Waning Immunity after the BNT162b2 Vaccine in Israel. *New England Journal of Medicine* **385**, e85 (2021).
16. Grannis, S. J. Interim Estimates of COVID-19 Vaccine Effectiveness Against COVID-19–Associated Emergency Department or Urgent Care Clinic Encounters and Hospitalizations Among Adults During SARS-CoV-2 B.1.617.2 (Delta) Variant Predominance — Nine States, June–August 2021. *MMWR Morb Mortal Wkly Rep* **70**, (2021).
17. Hansen, C. *et al.* Vaccine effectiveness against infection and COVID-19-associated hospitalisation with the Omicron (B.1.1.529) variant after vaccination with the BNT162b2 or mRNA-1273 vaccine: A nationwide Danish cohort study. Preprint at https://doi.org/10.21203/rs.3.rs-1486018/v1 (2024).
18. Hernandez-Avila, M. *et al.* Assessing the real-world effectiveness of five SARS-CoV-2 vaccines in a cohort of Mexican pensioners: a nationwide nested test-negative design study. *The Lancet Regional Health – Americas* **27**, (2023).
19. Irala, S. *et al.* COVID-19 vaccine effectiveness against hospitalizations in Paraguay, May 2021—April 2022: A test-negative design. *Vaccine* **41**, 6453–6460 (2023).

20. Katikireddi, S. V. *et al.* Two-dose ChAdOx1 nCoV-19 vaccine protection against COVID-19 hospital admissions and deaths over time: a retrospective, population-based cohort study in Scotland and Brazil. *The Lancet* **399**, 25–35 (2022).
21. Kirsebom, F. C. M. *et al.* Effectiveness of ChAdOx1-S COVID-19 booster vaccination against the Omicron and Delta variants in England. *Nat Commun* **13**, 7688 (2022).
22. Lauring, A. S. *et al.* Clinical severity of, and effectiveness of mRNA vaccines against, covid-19 from omicron, delta, and alpha SARS-CoV-2 variants in the United States: prospective observational study. *BMJ* **376**, e069761 (2022).
23. Lin, D.-Y. *et al.* Association of Primary and Booster Vaccination and Prior Infection With SARS-CoV-2 Infection and Severe COVID-19 Outcomes. *JAMA* **328**, 1415–1426 (2022).
24. Link-Gelles, R. *et al.* Estimation of COVID-19 mRNA Vaccine Effectiveness and COVID-19 Illness and Severity by Vaccination Status During Omicron BA.4 and BA.5 Sublineage Periods. *JAMA Network Open* **6**, e232598 (2023).
25. Lytras, T., Kontopidou, F., Lambrou, A. & Tsiodras, S. Comparative effectiveness and durability of COVID-19 vaccination against death and severe disease in an ongoing nationwide mass vaccination campaign. *Journal of Medical Virology* **94**, 5044–5050 (2022).
26. Mimura, W. *et al.* Effectiveness of BNT162b2 Against Infection, Symptomatic Infection, and Hospitalization Among Older Adults Aged ≥65 Years During the Delta Variant Predominance in Japan: The VENUS Study. *Journal of Epidemiology* **advpub**, (2023).
27. Monge, S. *et al.* Brand-specific vaccine effectiveness against SARS-CoV-2 infection, hospitalization and mortality, in people aged 50-59 years in Spain. *Revista Espanola de Salud Publica* **96**, (2022).
28. Nasreen, S. *et al.* Effectiveness of COVID-19 vaccines against symptomatic SARS-CoV-2 infection and severe outcomes with variants of concern in Ontario. *Nat Microbiol* **7**, 379–385 (2022).
29. Park, S. K. *et al.* Effectiveness of Heterologous COVID-19 Vaccine Booster in Korean Elderly Population, 2022. *J Korean Med Sci* **38**, e143 (2023).
30. Paternina-Caicedo, A. *et al.* Comparative effectiveness and duration of protection of ChAdOx1, CoronaVac, BNT162b2, mRNA-1273, and Ad26.COV2.S COVID-19 vaccines for symptomatic and hospitalized Mu, Delta, and Omicron: A test-negative case-control study. *Vaccine* **41**, 6291–6299 (2023).
31. Penayo, E. *et al.* Evaluation of vaccine effectiveness against COVID-19 in Paraguay, 2021. [in Spanish]. https://www.paho.org/es/node/86378 (2022).
32. Plumb, I. D. Effectiveness of COVID-19 mRNA Vaccination in Preventing COVID-19–Associated Hospitalization Among Adults with Previous SARS-CoV-2 Infection — United States, June 2021–February 2022. *MMWR Morb Mortal Wkly Rep* **71**, (2022).
33. Risk, M. *et al.* COVID-19 vaccine effectiveness against omicron (B.1.1.529) variant infection and hospitalisation in patients taking immunosuppressive medications: a retrospective cohort study. *The Lancet Rheumatology* **4**, e775–e784 (2022).
34. Sharma, A. *et al.* Effectiveness of Messenger RNA–based Vaccines During the Emergence of the Severe Acute Respiratory Syndrome Coronavirus 2 Omicron Variant. *Clinical Infectious Diseases* **75**, 2186–2192 (2022).
35. Skowronski, D. M. *et al.* Two-Dose Severe Acute Respiratory Syndrome Coronavirus 2 Vaccine Effectiveness With Mixed Schedules and Extended Dosing Intervals: Test-Negative Design Studies From British Columbia and Quebec, Canada. *Clinical Infectious Diseases* **75**, 1980–1992 (2022).
36. Šmíd, M. *et al.* Protection by Vaccines and Previous Infection Against the Omicron Variant of Severe Acute Respiratory Syndrome Coronavirus 2. *The Journal of Infectious Diseases* **226**, 1385–1390 (2022).
37. Starrfelt, J. *et al.* Age and product dependent vaccine effectiveness against SARS-CoV-2 infection and hospitalisation among adults in Norway: a national cohort study, July–November 2021. *BMC Medicine* **20**, 278 (2022).
38. Stowe, J. *et al.* Effectiveness of COVID-19 vaccines against hospital admission with the Delta (B.1.617.2) variant. Preprint at https://khub.net/web/phe-national/public-library/-/document_library/v2WsRK3ZlEig/view_file/479607329?_com_liferay_document_library_web_

portlet_DLPortlet_INSTANCE_v2WsRK3ZlEig_redirect=https%253A%252F%252Fkhub.net%253A443%252Fweb%252Fphe-national%252Fpublic-library%252F-%25 (2021).
39. Stowe, J. *et al.* Effectiveness of COVID-19 vaccines against Omicron and Delta hospitalisation, a test negative case-control study. *Nat Commun* **13**, 5736 (2022).
40. Tang, P. *et al.* BNT162b2 and mRNA-1273 COVID-19 vaccine effectiveness against the SARS-CoV-2 Delta variant in Qatar. *Nat Med* **27**, 2136–2143 (2021).
41. Tartof, S. Y. *et al.* Durability of BNT162b2 vaccine against hospital and emergency department admissions due to the omicron and delta variants in a large health system in the USA: a test-negative case–control study. *The Lancet Respiratory Medicine* **10**, 689–699 (2022).
42. Tartof, S. Y. *et al.* Effectiveness of mRNA BNT162b2 COVID-19 vaccine up to 6 months in a large integrated health system in the USA: a retrospective cohort study. *The Lancet* **398**, 1407–1416 (2021).
43. Tartof, S. Y. *et al.* BNT162b2 vaccine effectiveness against SARS-CoV-2 omicron BA.4 and BA.5. *The Lancet Infectious Diseases* **22**, 1663–1665 (2022).
44. Tartof, S. Y. *et al.* Effectiveness and durability of BNT162b2 vaccine against hospital and emergency department admissions due to SARS-CoV-2 omicron sub-lineages BA.1 and BA.2 in a large health system in the USA: a test-negative, case-control study. *The Lancet Respiratory Medicine* **11**, 176–187 (2023).
45. Tseng, H. F. *et al.* Effectiveness of mRNA-1273 against SARS-CoV-2 Omicron and Delta variants. *Nat Med* **28**, 1063–1071 (2022).
46. Tsundue, T. *et al.* First and second doses of Covishield vaccine provided high level of protection against SARS-CoV-2 infection in highly transmissible settings: results from a prospective cohort of participants residing in congregate facilities in India. *BMJ Global Health* **7**, e008271 (2022).
47. Vokó, Z. *et al.* Effectiveness and Waning of Protection With Different SARS-CoV-2 Primary and Booster Vaccines During the Delta Pandemic Wave in 2021 in Hungary (HUN-VE 3 Study). *Frontiers in Immunology* **13**, (2022).
48. Wan, E. Y. F. *et al.* Effectiveness of BNT162b2 and CoronaVac vaccinations against SARS-CoV-2 omicron infection in people aged 60 years or above: a case–control study. *Journal of Travel Medicine* **29**, taac119 (2022).
49. Yan, V. K. C. *et al.* Effectiveness of BNT162b2 and CoronaVac vaccinations against mortality and severe complications after SARS-CoV-2 Omicron BA.2 infection: a case–control study. *Emerging Microbes & Infections* **11**, 2304–2314 (2022).
50. Yan, V. K. C. *et al.* Waning effectiveness against COVID-19-related hospitalization, severe complications, and mortality with two to three doses of CoronaVac and BNT162b2: a case–control study. *Emerging Microbes & Infections* **12**, 2209201 (2023).

Supplementary Methods references

1. Wu, N. et al. (Supplementary appendix) Long-term effectiveness of COVID-19 vaccines against infections, hospitalisations, and mortality in adults: findings from a rapid living systematic evidence synthesis and meta-analysis up to December, 2022. The Lancet Respiratory Medicine 11, 439–452 (2023).
2. Higgins JPT, Thomas J, Chandler J, Cumpston M, Li T, Page MJ, Welch VA (editors). Cochrane Handbook for Systematic Reviews of Interventions version 6.4 (updated August 2023). Cochrane, 2023. www.training.cochrane.org/handbook.
3. Harrer, M., Cuijpers, P., Furukawa, T. A. & Ebert, D. D. Doing Meta-Analysis with R: A Hands-On Guide. (Chapman and Hall/CRC, Boca Raton, 2021). doi:10.1201/9781003107347.
