# Supplementary material for: Global disparities in the introduction, scale-up, and effectiveness evaluation of COVID-19 vaccines
Source: Nat Commun. 2025 Oct 13;16:9059. doi: 10.1038/s41467-025-63950-w (PMC12518578; doi:10.1038/s41467-025-63950-w)
Supplement: Supplementary file 3 — Description of Additional Supplementary Files [file 41467_2025_63950_MOESM3_ESM.docx]

**Description of Additional Supplementary Files**

File Name: Supplementary Data 1
Description: Country-/territory-level data on income status, vaccine introduction, vaccine scale-up, and evaluation of vaccine effectiveness.

File Name: Supplementary Data 2
Description: Vaccine effectiveness estimates used for descriptive analyses and metaregression.
